# A web-based predictive model for overall survival of patients with cutaneous Merkel cell carcinoma: A population-based study

**DOI:** 10.3389/fendo.2022.1038181

**Published:** 2022-11-24

**Authors:** Wen Xu, Yijun Le, Jianzhong Zhang

**Affiliations:** ^1^ Department of Dermatology, Peking University People’s Hospital, Beijing, China; ^2^ Musculoskeletal Tumor Center, Peking University People’s Hospital, Beijing, China

**Keywords:** merkel cell carcinoma, overall survival, web-based nomogram, SEER, online application

## Abstract

**Background:**

Merkel cell carcinoma (MCC) is an aggressive neuroendocrine carcinoma with a high mortality rate, so it is necessary to create models to predict overall survival of MCC. We developed an easy-to-use web-based calculator to predict the OS of MCC patients based on the nomogram.

**Methods:**

MCC patients between 2004 and 2015 were collected from the Surveillance, Epidemiology, and End Results (SEER) database and randomly assigned to training and validation cohorts. Patients between 2016-2017 serve as the external validation cohort. Relevant risk factors were identified by univariate and multivariate COX hazards regression methods and combined to produce nomograms. The concordance index (C-index), area under the receiver operating characteristic (AUC) curve, and calibration plots have demonstrated the predictive power of the nomograms. Decision curve analysis (DCA) was used to measure nomograms in clinical practice. Patients were divided into three groups according to the scores of the nomogram.

**Results:**

A total of 3480 patients were randomly assigned to the training group and validation group in this study. Meaningful prognostic factors were applied to the establishment of nomograms. The C-index for OS was 0.725 (95% CI: 0.706-0.741) in the training cohort and 0.710 (95% CI: 0.683-0.737) in the validation cohort. In the external validation cohort, C-index was 0.763 (95% CI: 0.734–0.792). The C-index of training cohort, validation cohort and external validation cohort for CSS were 0.743 (95% CI:0.725-0.761), 0.739(95%CI:0.712-0.766) and 0.774 (95%CI:0.735-0.813), respectively. The AUC and calibration plots of 1-, 3-, and 5-year OS rates showed that the nomogram had good predictive power. DCA demonstrated that the nomogram constructed in this study could provide a clinical net benefit. Our calculator demonstrated excellent predictive capabilities for better risk grouping of MCC patients.

**Conclusion:**

We created novel nomograms of prognostic factors for MCC, which more accurately and comprehensively predicted 1-, 3-, and 5-year OS/CSS in MCC patients. We established a calculator which can easily and quickly calculate the risk grouping of MCC patients by inputting clinically relevant characteristics. This can help clinicians identify high-risk patients as early as possible, carry out personalized treatment, follow-up, and monitoring, and improve the survival rate of MCC patients.

## Introduction

Merkel cell carcinoma (MCC) is a rare, aggressive neuroendocrine carcinoma (1). MCC has been hypothesized to originate from Merkel cell precursors (potentially derived from epidermal stem cells or hair follicle stem cells), pre-B cells, pro-B cells, or dermal fibroblasts (1). Most MCCs present as rapidly growing red or violaceous firm nodules on the sun-exposed skin of the aged (2). MCC carcinogenesis can be initiated by the clonal integration of the Merkel cell polyomavirus (MCPyV) genome or UV-mediated DNA damage caused by chronic exposure to sunlight (1). In addition, immunosuppressive status (1), chronic arsenic exposure (3), and chronic inflammation (4) have also been identified as risk factors for inducing MCC. Therapeutic approaches against MCC include surgery, radiotherapy, chemotherapy, immunotherapy, and targeted molecular therapy (5). In general, surgery is the first-line treatment for primary MCC. For metastatic MCC, the effectiveness of immunotherapy has been validated and becomes the current first choice (5).The incidence of MCC increases exponentially, ranging from 0.1 (per 100,000 person-years) in individuals ages 40 to 44, to 1.0 in those ages 60 to 64, and to 9.8 in those older than age 85 (6). Overall survival at 5 years is approximately 51% for local disease, 35% for nodal disease, and 14% for distant disease (2). Considering the high mortality rate of MCC, it is necessary to summarize the prognostic factors. Prognostic risk factors that have been reported include male gender, advanced age, immunosuppressed status, MCPyV negativity, low CD8+ T cell levels, lymphovascular invasion, tumor growth pattern, lymph node number, and stage, etc (7–14). Risk stratification of MCC patients allows for better monitoring and management. This has important implications for improving the prognosis of MCC.

A nomogram is a user-friendly mathematical model based on the COX proportional risk regression model that uses known clinical and pathological characteristics to predict the probability of an event, which not only does not sacrifice the accuracy of the regression model, but also adds the excellent features of user-friendliness and ease of use.

## Materials and methods

### Data source and selection of variables

The original data in this study were extracted from the SEER database, one of the largest oncology databases available to the public, covering approximately 28% of the US population. The SEER database agreement has been signed and provided permission to access SEER information (accession username: 12906-Nov2021), so we were able to obtain patient demographics, tumor characteristics, and survival status from the SEER database. Since the SEER database is accessible to the public, institutional review board approval or informed consent was not required for our study. And all the methods used in our study also comply with the rules of the SEER database.

**Figure 1:**
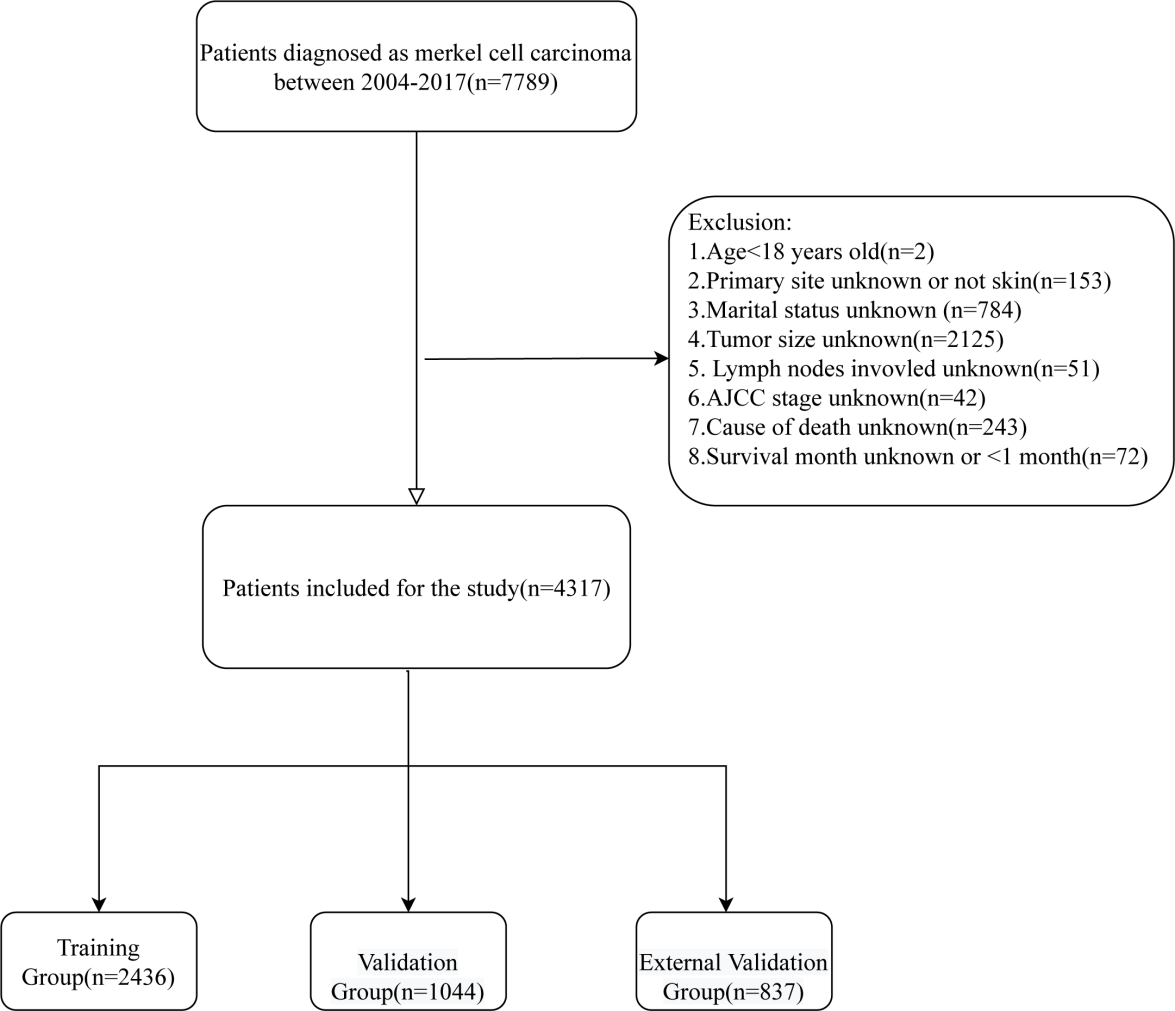
Flow chart for inclusion and exclusion of patients.

All patients with cutaneous Merkel cell carcinoma diagnosed between 2004 and 2017 were taken into account for this study, and the exclusion criteria were (1): Age<18 years old (2); Primary site unknown or not skin (3). Marital status unknown (4); Tumor size unknown (5); Lymph nodes involvement unknown; (6) AJCC stage unknown; (7) Cause of death unknown; (8) Survival month unknown or <1 month. At last, 4317 patients were included in this study. By using the SEER*Stat 8.4.0 (http://seer.cancer.gov//seerstat/), the demographic and clinical characteristics including age, gender, marital status, primary site, multiple primary tumors, tumor size, lymph nodes involvement, AJCC stage, surgery, radiotherapy, chemotherapy, cause of death, survival status, and survival time were obtained for these patients. The screening flow chart is shown in **Figure 1**.

### Statistics analysis

All statistical analysis in our study was performed with R software version 4.1.3(https://www.r-project.org/). “survival”, “rms”, “ROCR”, “ggDCA”, “DynNom” and “shiny” R packages were used to construct and validate the nomograms, plot the ROC curves, formulate the calibration curves and establish DCA. The result is considered statistically significant when the P value is less than 0.05(two-sided).

Using R software, patients between 2004-2015 were randomly divided into training and validation groups in a 7:3 ratio and the association between the two groups was compared using a chi-square test. Moreover, patients between 2016-2017 serve as the external validation cohort. The Cox proportional-hazards risk model was used for univariate and multivariate analysis to identify independent risk factors for MCC. The independent predictors were then used to create a nomogram. This nomogram can be used to predict 1-, 3-, and 5-year overall survival rates of MCC patients. The receiver operating characteristic (ROC), the area under the curve (AUC), calibration curves (bootstrap=1,000 resampling validation),and C-index were used to assess the predictive power of the model. Decision curve analysis was used to evaluate the clinical value of the nomogram. DCA is a new algorithm to assess the clinical utility value of the column line graph by estimating the net benefit at each risk threshold. The Nomogram for CSS was subsequently created and validated in the same way. Finally, patients were divided into three groups according to the scores of the nomogram: low risk, intermediate risk, and high risk, and the Kaplan-Meier curve and log-rank test were used to compare the differences between the three groups.

## Results

### Demographic and clinicopathological characteristics

After screening, 4317 patients were diagnosed with MCC, of which patients between 2004-2015 (n=3480) were used to establish and internally validate the prediction model. The demographic and clinical characteristics of the training group and validation group are shown in **Table 1**. The mean age of these patients was 75.2 years, predominantly elderly, 2199 (63.2%) patients were male, 3327 (95.6%) patients were white, 2178 (62.6%) patients were married, and the primary sites were concentrated in the head, neck, face and extremities, 1482 (42.6%), 1590 (45.7%), respectively.1971 (56.6%) patients had no other primary tumors. 2160 (62.1%) patients whose tumor size was <2 cm and 2491 (71.6%) patients had no lymph node involvement. 1653 (47.5%), 688 (19.8%), 951 (27.3%), and 188 (5.4%) patients had AJCC stage I, II, III, and IV, respectively. 3282 (94.3%) patients underwent surgery, 1874 (53.9%) patients received radiotherapy, and the majority of patients (n=3112 (89.4%)) did not receive radiotherapy. The mean survival time was 51.1 months. At the endpoint, 2157 (62.0%) patients died, of which 947 (27.2%) died of MCC. there was no significant difference between the training and validation groups (all P values > 0.05).

**Table 1:** The demographics and clinical features of patients with merkel cell carcinoma in different cohorts.

	Training group (N=2436)	Validation group (N=1044)	Overall (N=3480)	P-value
**Age**	74.9 (11.3)	75.7 (11.4)	75.2 (11.3)	0.576
**Sex**				
Female	920 (37.8%)	361 (34.6%)	1281 (36.8%)	0.203
Male	1516 (62.2%)	683 (65.4%)	2199 (63.2%)	
**Race**				
White	2322 (95.3%)	1005 (96.3%)	3327 (95.6%)	0.78
Black	39 (1.6%)	15 (1.4%)	54 (1.6%)	
Other	75 (3.1%)	24 (2.3%)	99 (2.8%)	
**Marital status**				
Married	1511 (62.0%)	667 (63.9%)	2178 (62.6%)	0.854
Single	218 (8.9%)	77 (7.4%)	295 (8.5%)	
Divorced or Separated	175 (7.2%)	72 (6.9%)	247 (7.1%)	
Widowed	532 (21.8%)	228 (21.8%)	760 (21.8%)	
**Primary site**				
Head Neck and Face	1029 (42.2%)	453 (43.4%)	1482 (42.6%)	0.847
Extremity	1122 (46.1%)	468 (44.8%)	1590 (45.7%)	
Trunk	274 (11.2%)	114 (10.9%)	388 (11.1%)	
Skin, NOS	11 (0.5%)	9 (0.9%)	20 (0.6%)	
**Multiple primary tumors**				
No	1381 (56.7%)	590 (56.5%)	1971 (56.6%)	0.995
Yes	1055 (43.3%)	454 (43.5%)	1509 (43.4%)	
**Tumor size**				
≤2cm	1510 (62.0%)	650 (62.3%)	2160 (62.1%)	0.619
2-5cm	725 (29.8%)	324 (31.0%)	1049 (30.1%)	
>5cm	201 (8.3%)	70 (6.7%)	271 (7.8%)	
**Lymph nodes involved**				
No	1750 (71.8%)	741 (71.0%)	2491 (71.6%)	0.875
Yes	686 (28.2%)	303 (29.0%)	989 (28.4%)	
**AJCC stage**				
I	1154 (47.4%)	499 (47.8%)	1653 (47.5%)	0.963
II	493 (20.2%)	195 (18.7%)	688 (19.8%)	
III	656 (26.9%)	295 (28.3%)	951 (27.3%)	
IV	133 (5.5%)	55 (5.3%)	188 (5.4%)	
**Surgery**				
No	131 (5.4%)	67 (6.4%)	198 (5.7%)	0.479
Yes	2305 (94.6%)	977 (93.6%)	3282 (94.3%)	
**Radiotherapy**				
No	1117 (45.9%)	489 (46.8%)	1606 (46.1%)	0.867
Yes	1319 (54.1%)	555 (53.2%)	1874 (53.9%)	
**Chemotherapy**				
No	2167 (89.0%)	945 (90.5%)	3112 (89.4%)	0.391
Yes	269 (11.0%)	99 (9.5%)	368 (10.6%)	
**Survival months**	51.1 (42.9)	51.2 (41.5)	51.1 (42.5)	0.247
**Overall survival**				
Alive	921 (37.8%)	402 (38.5%)	1323 (38.0%)	0.927
Dead	1515 (62.2%)	642 (61.5%)	2157 (62.0%)	
**Cancer-specific survival**				
Alive	1772 (72.7%)	761 (72.9%)	2533 (72.8%)	0.996
Dead	664 (27.3%)	283 (27.1%)	947 (27.2%)	

### Univariate and multivariate cox regression analysis

We used univariate regression analysis to identify eleven risk factors associated with OS, including age, sex, race, marital status, primary site, multiple primary tumors, tumor size, lymph nodes involvement, AJCC stage, surgery, radiotherapy, and chemotherapy. We then performed a multivariate regression analysis using above-selected clinical characteristics to identify the independent risk factors for OS (**Table 2**). The results showed that the variables including age, sex, race, primary site, multiple primary tumors, tumor size, lymph nodes involvement, AJCC stage, surgery, and radiotherapy were the independent risk factors predicting OS in MCC patients. In the same way, we found that age, sex, primary site, tumor size, lymph nodes involvement, and AJCC stage were independent risk factors associated with CSS in MCC patients (**Table 3**).

**Table 2:** Univariate and multivariate Cox regression analysis of OS.

	Univariate			Multivariate		
	HR	95%CI	P	HR	95%CI	P
**Age**	1.06	1.05-1.06	<0.001	1.06	1.05 - 1.06	<0.001
**Sex**						
Female						
Male	1.42	1.28-1.58	<0.001	1.49	1.33 - 1.68	<0.001
**Race**						
White						
Black	1.27	0.86-1.87	0.23	1.25	0.85 - 1.86	0.26
Other	0.54	0.38-0.77	<0.001	0.6	0.42 - 0.86	0.01
**Marital status**						
Married						
Single	0.78	0.7-0.86	<0.001	1.04	0.86 - 1.26	0.68
Divorced or Separated	1	0.85-1.18	0.98	1.17	0.94 - 1.46	0.17
Widowed	1.4	0.73-2.71	0.31	1.13	0.99 - 1.29	0.07
**Primary site**						
Head Neck and Face						
Extremity	0.97	0.8-1.17	0.73	0.89	0.8 - 1	0.05
Trunk	0.86	0.7-1.07	0.18	1.07	0.9 - 1.27	0.46
Skin, NOS	1.59	1.42-1.79	<0.001	1.04	0.54 - 2.02	0.91
**Multiple primary tumors**						
No						
Yes	1.3	1.18-1.44	<0.001	1.17	1.06 - 1.3	<0.001
**Tumor size**						
≤2cm						
2-5cm	1.46	1.31-1.63	<0.001	1.28	1.07 - 1.53	0.01
>5cm	1.73	1.45-2.06	<0.001	1.59	1.28 - 1.99	<0.001
**Lymph nodes involved**						
No						
Yes	1.69	1.52-1.88	<0.001	1.41	1.09 - 1.82	0.01
**AJCC stage**						
I						
II	1.36	1.18-1.55	<0.001	1.04	0.83 - 1.29	0.76
III	1.73	1.53-1.95	<0.001	1.24	0.94 - 1.64	0.13
IV	4.98	4.08-6.07	<0.001	3.06	2.3 - 4.07	<0.001
**Surgery**						
No						
Yes	0.57	0.47-0.7	<0.001	0.76	0.61 - 0.94	0.01
**Radiotherapy**						
No						
Yes	0.75	0.68-0.83	<0.001	0.74	0.67 - 0.82	<0.001
**Chemotherapy**						
No						
Yes	1.32	1.13-1.53	<0.001	1.13	0.95 - 1.35	0.17

**Table 3:** Univariate and multivariate Cox regression analysis of CSS.

	Univariate			Multivariate		
	HR	95%CI	P	HR	95%CI	P
**Age**	1.02	1.02-1.03	<0.001	1.03	1.02-1.04	<0.001
**Sex**						
Female						
Male	1.73	1.46-2.05	<0.001	1.63	1.36-1.95	<0.001
**Race**						
White						
Black	1.64	0.98-2.74	0.06			
Other	0.6	0.35-1.01	0.06			
**Marital status**						
Married						
Single	1.08	0.83-1.41	0.58	1.1	0.84-1.43	0.50
Divorced or Separated	0.85	0.62-1.18	0.34	1.07	0.77-1.5	0.67
Widowed	1.23	1.03-1.49	0.03	1.14	0.93-1.4	0.20
**Primary site**						
Head Neck and Face						
Extremity	0.74	0.63-0.87	<0.001	0.72	0.61-0.86	<0.001
Trunk	1.24	0.98-1.56	0.07	0.91	0.72-1.17	0.47
Skin, NOS	1.82	0.75-4.39	0.19	1.45	0.59-3.53	0.42
**Multiple primary tumors**						
No						
Yes	0.9	0.77-1.05	0.18			
**Tumor size**						
≤2cm						
2-5cm	1.79	1.52-2.12	<0.001	1.29	1.03-1.62	0.03
>5cm	3.01	2.4-3.78	<0.001	2.04	1.55-2.69	<0.001
**Lymph nodes involved**						
No						
Yes	3.43	2.95-4	<0.001	1.81	1.3-2.53	<0.001
**AJCC stage**						
I						
II	1.45	1.14-1.86	0.003	1.03	0.75-1.43	0.85
III	3.63	3.01-4.37	<0.001	1.71	1.16-2.52	0.006
IV	11.96	9.28-15.41	<0.001	5.58	3.77-8.25	<0.001
**Surgery**						
No						
Yes	0.49	0.37-0.65	<0.001	0.9	0.67-1.22	0.50
**Radiotherapy**						
No						
Yes	1.04	0.9-1.22	0.58			
**Chemotherapy**						
No						
Yes	2.44	2.02-2.95	<0.001	1.18	0.95-1.47	0.14

### Construction and validation of the nomogram

**Figure 2:**
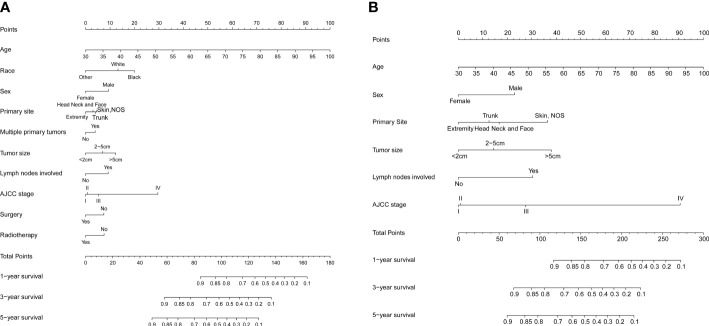
Nomograms predicting 1-, 3-, and 5-year OS **(A)** and CS **(B)** of patients with MCC.

Significant independent risk factors from the multivariate analysis were used to construct the nomograms to predict 1-, 3-, and 5-year OS (**Figure 2A**) and CSS (**Figure 2B**). The scale at the top of the nomogram provides a score for each prognostic variable, and the sum of all scores corresponds to the scale at the bottom of the nomogram for the nomogram display of OS/CSS prediction. The nomograms were then validated by C-index, calibration curves, and ROC curves.

**Figure 3:**
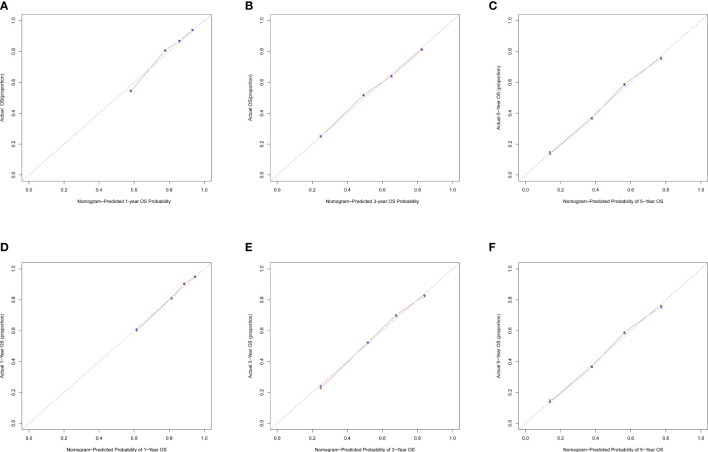
Calibration curves of nomogram. **(A)** For 1-year OS in training cohort; **(B)** For 3-year OS in training cohort; **(C)** For 5-year OS in training cohort; **(D)** For 1-year OS in validation cohort; **(E)** for 3-year OS in validation cohort; **(F)** for 5-year OS in validation cohort.

**Figure 4:**
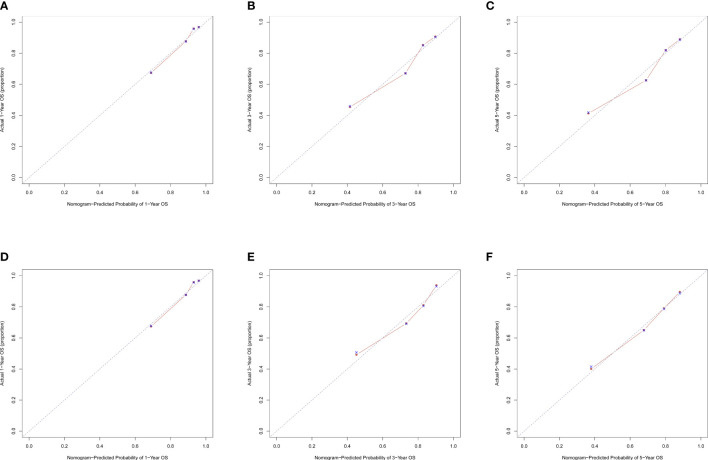
Calibration curves of nomogram. **(A)** For 1-year CSS in training cohort; **(B)** For 3-year CSS in training cohort; **(C)** For 5-year CSS in training cohort; **(D)** For 1-year CSS in validation cohort; **(E)** for 3-year CSS in validation cohort; **(F)** for 5-year CSS in validation cohort.

**Figure 5:**
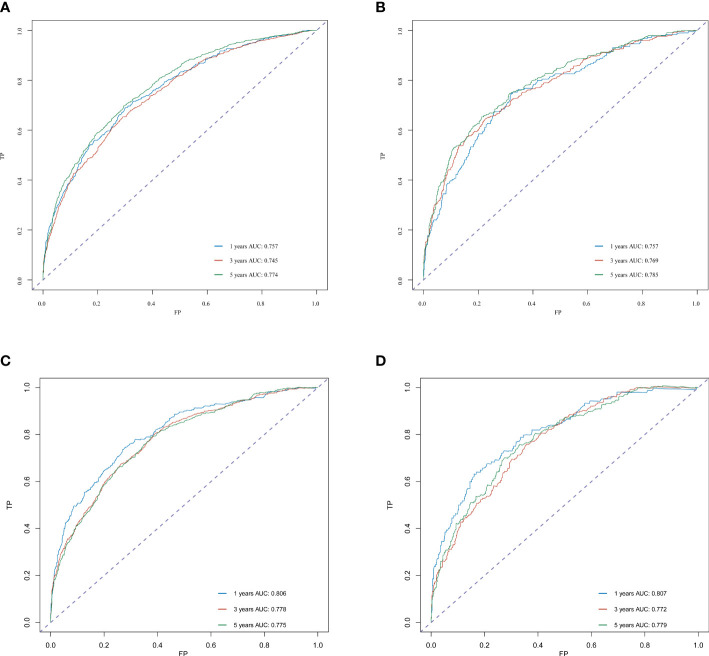
ROC curve analysis to predict 1-, 3- and 5-year OS and CSS rates in merkel cell carcinoma Patients. **(A)** ROC curves for OS in the training cohort. **(B)** ROC curves for OS in the validation cohort. **(C)** ROC curves for CSS in the training cohort. **(D)** ROC curves for CSS in the validation cohort. AUC, area under the curve; ROC, receiver operating characteristic; TP, true positive rates; FP, false positive rate.

**Figure 6:**
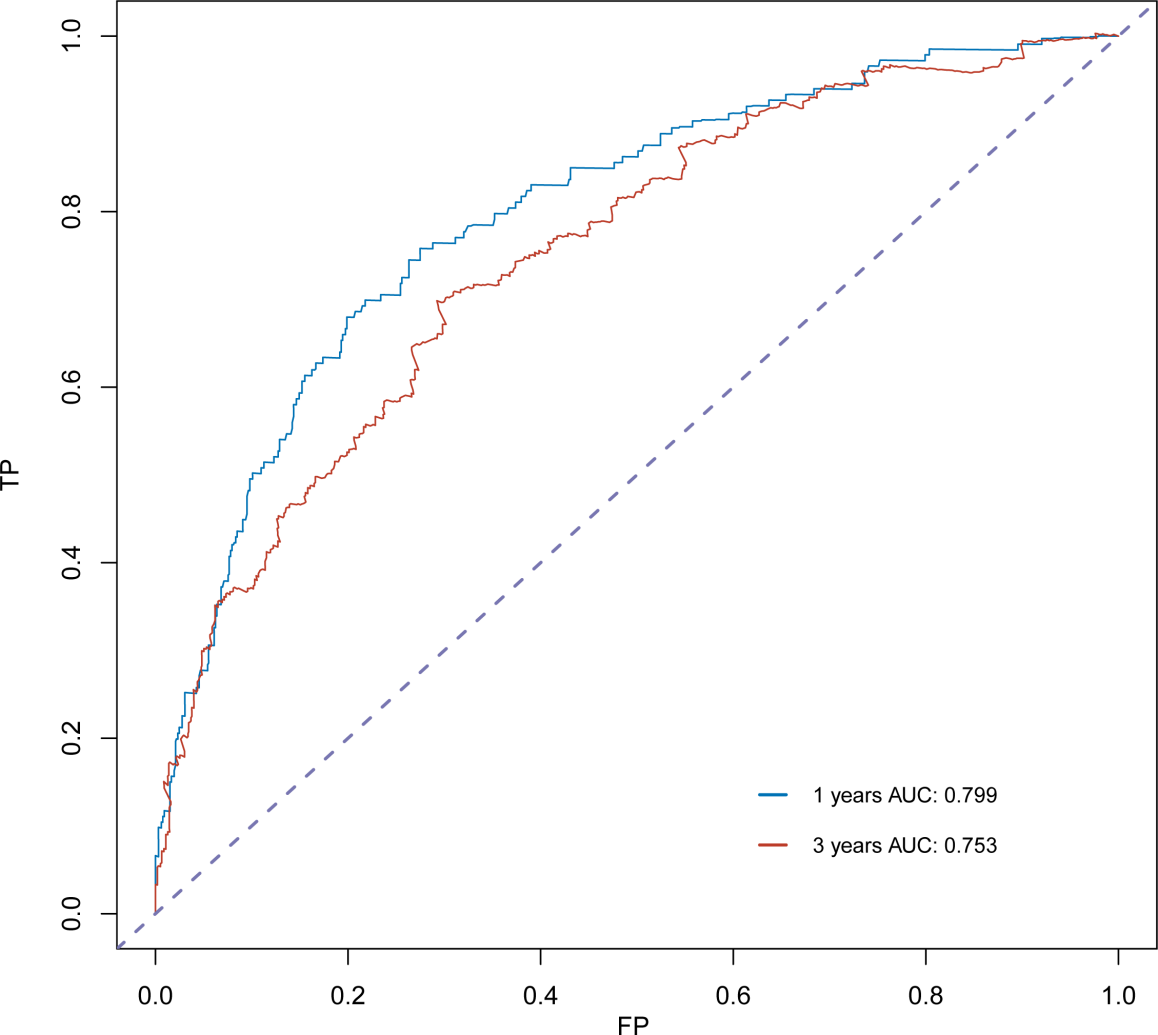
ROC curve analysis to predict 1- and 3-year OS rates in the external validation cohort.

**Figure 7:**
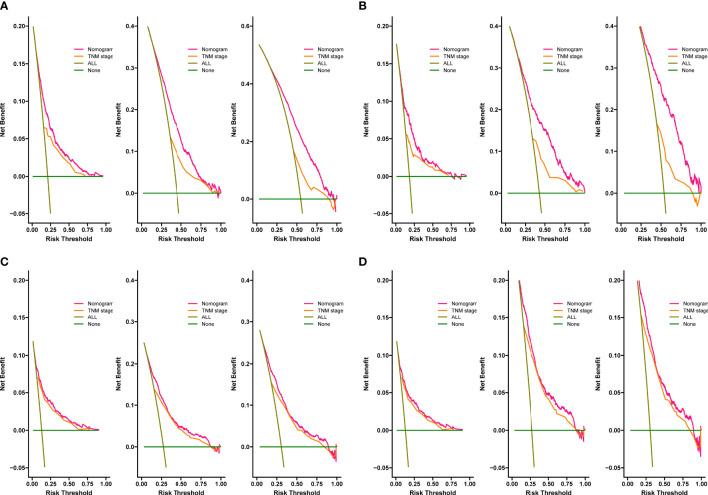
Decision curves of the nomogram predicting OS in training cohort **(A)** and validation cohort **(B)**. Decision curves of the nomogram predicting CSS in training cohort **(C)** and validation cohort **(D)**. The y-axis represents the net benefit, and the x-axis represents the threshold probability.

**Figure 8:**
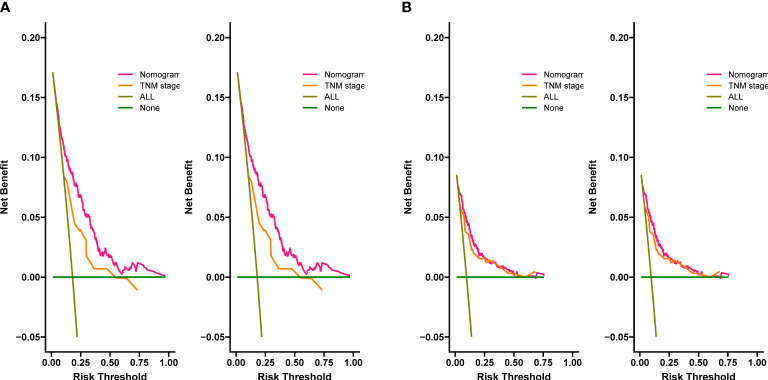
Decision curves of the nomogram predicting OS in external validation cohort **(A)**, the nomogram predicting CSS in external validation cohort **(B)**.

The C-index for OS in the training cohort was 0.725 (95% CI: 0.706-0.741), while the c-index for OS in the validation cohort was 0.710 (95% CI: 0.683-0.737). In the external validation cohort, C-index was 0.763 (95% CI: 0.734–0.792). The C-index of training cohort, validation cohort and external validation cohort for CSS were 0.743 (95% CI:0.725-0.761), 0.739(95%CI:0.712-0.766) and 0.774 (95%CI:0.735-0.813), respectively. The calibration curves of the training and validation cohorts used to predict OS showed good agreement between the observed and predicted results (**Figure 3**). Also, the calibration curves for predicting patient CSS were performed accurately (**Figure 4**). In the training cohort, the AUCs for predicting 1-year, 3-year, and 5-year OS were 0.757,0.745, and 0.774 (**Figure 5A**), respectively. And the AUCs for predicting 1-year, 3-year, and 5-year OS in the validation cohort were 0.757,0.769, and 0.785 (**Figure 5B**), respectively. For predicting CSS, the AUCs for 1-year-, 3-year-, and 5-year in the training cohort were 0.806, 0.778, 0.775 (**Figure 5C**), and 0.807,0.772, 0.779 (**Figure 5D**) in the validation cohort. The AUCs of predicted 1-year, and 3-year OS in the external validation cohort were 0.799,0.753 (**Figure 6**), respectively. The DCAs of the training cohort and the validation cohort showed that the clinical application of the nomogram was superior to that of the existing TNM staging system (**Figure 7**), and the DCAs of the external validation cohort performed equally well (**Figure 8**).

### Risk stratification and online application for predicting OS

**Figure 9:**
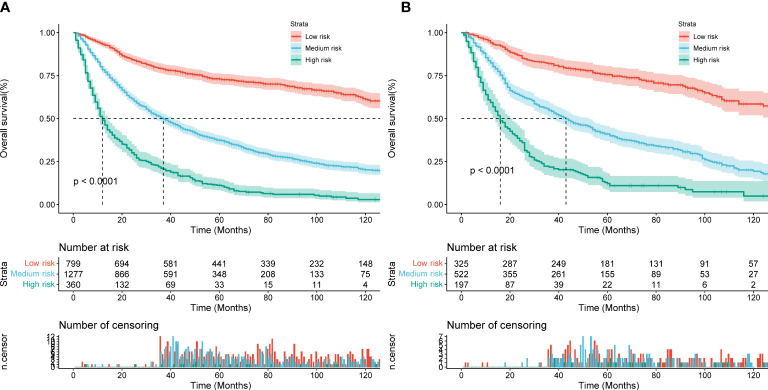
Kaplan–Meier curves of OS for patients in the low-, medium- and high-risk groups in the training Cohort **(A)** and validation Cohort **(B)**.

Based on the total score of the patients derived from the nomogram, we created a risk stratification system. Each patient was divided into three groups: low-risk, intermediate-risk, and high-risk groups. Kaplan-Meire analysis curves showed that the low-risk group had the best prognosis, the intermediate-risk group the second best, and the high-risk group the worst prognosis (**Figure 9**). Finally, we developed an easy-to-use web-based calculator to predict the OS of MCC patients based on the nomogram, which can be accessed at https://yijunle.shinyapps.io/DynNomapp/. The probability of survival at the predicted time can be obtained by entering the patient’s characteristics in the web page. This calculator is very convenient for clinical use.

## Discussion

Merkel cell carcinoma (MCC) is a rare, neuroendocrine, cutaneous malignancy that was first described in 1972 (15). 65% of MCCs present with local disease and no clinical or pathologic evidence of metastasis to regional lymph nodes (LNs) or distant sites (2). Approximately 5% of MCC are found in the LNs without a primary tumor on the skin (2). 5-year OS ranging from 51% for patients with local disease to 14% for patients with distant metastases (2). MCC recurs most often within the first 2 years after diagnosis (16). The monitoring of the first two years is particularly important. To better manage and monitor MCC patients, we believe that summarization of prognostic factors and risk stratification is necessary.

In this study, based on univariate and multivariate cox proportional hazards regression analysis, we found that age, gender, race, primary site, multiple tumors, tumor size, lymph node involvement, stage, surgery, and radiotherapy were all prognostic risk factors for OS rate in MCC patients. Age, gender, primary site, tumor size, lymph node involvement, and stage were all prognostic risk factors for CSS rate in MCC patients. At the same time, we created nomograms to quantitatively predict the 1-, 3-, and 5-year OS rates and CSS rates of different individuals through the characteristics of MCC. Additionally, MCC patients have an increased risk of hematologic malignancies and developing secondary malignancies, both affecting OS (17). Therefore, in this study, the OS rate was used to divide the risk group. Through the nomogram of the OS rate, the risk scores of all MCC patients were calculated, and the patients were divided into low-risk, intermediate-risk, and high-risk groups. We made a calculator that inputs the patient’s personal basic information and can quickly calculate the patient’s risk score. Through our calculator, high-risk patients with MCC can be better identified, which is helpful for follow-up management and monitoring, and can better improve the survival rate of MCC patients.

Previous studies have confirmed that advanced age affects the survival rate of MCC patients, and this was also verified in our study (8). The incidence of MCC increases with age, the incidence rate reported in patients older than 85 years was even higher with a peak incidence rate of 17.6 (17). This may be related to long-term UV exposure and increased complications in old age. Morbidity and mortality were higher in males than in females, which is consistent with our study (18, 19). In the present study, the race was shown to be one of the prognostic factors for OS, but not in CSS. Skin pigmentation seems to protect against MCC, as black individuals have a considerably lower risk of MCC than white populations (1). The majority of the population included in this study were Caucasians, which was related to the limited statistical population in the SEER database. Our study showed that blacks have the worse prognosis, but the lack of a large amount of data from other races may lead to some bias in the research results. We still need larger populations and more comprehensive ethnic data to analyze the relationship between MCC and ethnicity. MCC is located mostly on sun-exposed areas, particularly the head and neck and also, less frequently, the extremities and buttocks (1). The primary site has always been considered to be an important factor affecting prognosis. It has been previously reported that tumor localization in the head and neck has an adverse effect on survival (20). In this study, for OS, we found that MCC of the trunk had the best prognosis, followed by the extremities, and the head and neck had the worse prognosis, while other sites had the worst prognosis. However, for CSS, MCC of the extremities had the best prognosis, followed by the head and neck, and the trunk had the worse prognosis, while other sites had the worst prognosis. This is slightly different from previous research. Multiple lesions are rare in MCC, and previous reports rarely mention the impact of multiple tumors on prognosis (1). We found that multiple tumors may lead to a worse prognosis.

Tumor size is also an important factor affecting prognosis. The 5-year survival rate decreased gradually with the tumor size (2). This is the same result as our study and we found that tumor size had a greater impact on CSS than OS. Our study showed that lymph nodes involvement is an important factor affecting the prognosis of MCC. A previous study demonstrated that pathological nodal staging more precisely predicts survival compared to clinical nodal staging (2). Meanwhile, patients with the occult nodal disease appear to have a better prognosis than those with clinically detected nodal disease (13). This likely reflects active immune clearance of the primary tumor prior to diagnosis (21). Patients with primary skin tumor lesions and lymph node involvement have a worse prognosis. At the same time, consistent with the results of the previous study, the higher the SEER stage, the worse the prognosis (22). That is, surrounding tissue invasion, lymph node metastasis and distant metastasis were all poor prognostic factors for MCC.

The treatment of MCC depends on the pathologic characteristics of the primary tumor and the extent of the disease, particularly the presence or absence of involved LNs or distant metastases (23). The presence or absence of metastasis will influence the choice of MCC treatment. Surgery remains the most common method by which primary MCC tumors are removed (5). Patients whose tumors cannot be completely excised, who are not surgical candidates, or refuse surgery may receive radiation treatment in its place (5). Chemotherapy offers modest benefits and is too toxic to be generally preferred (24). Anti-PD-1 antibodies, such as avelumab, and pembrolizumab, have received FDA approval for use in patients with locally advanced or metastatic MCC (25–27). In addition, anti-CTLA-4 monoclonal antibody and adoptive T cell or natural killer cell transfer are both possible methods to treat MCC (1, 5). Immunotherapy has a good efficacy and safety profile, and it has now become the standard-of-care for metastatic MCC. As the understanding of MCC has deepened, targeted molecular therapy and vaccination are in gradual development, which are potential options to treat and prevent MCC (28–30). This study also further confirmed that surgery and radiotherapy can achieve a higher OS rate. This is the same result as previously reported (8). Meanwhile, we did not find chemotherapy to significantly improve outcomes. After 2018, with the introduction of immunotherapy and targeted molecular therapy, the survival rate of MCC patients has improved. At present, there are still more immunotherapy and targeted molecular therapy in research. Since the 5-year survival rate is set as the observation endpoint in this study, we only obtained population samples before 2018 for research, and only used surgery, radiotherapy and chemotherapy as prognostic risk factors for analysis. In this study, we lack data on immunotherapy and targeted molecular therapy, which is related to the limitations of the database and our population selection. This is one of the limitations of this study.

In this study, we included prognostic-related characteristics, such as age, gender, race, marital status, primary site, multiple tumors, tumor size, lymph nodes involvement, AJCC stage, and treatments, through the large population data of the SEER database. These factors are readily available in the clinic and can better assess the risk of MCC patients. In the present study, the internal C-index was above 0.7 and the external C-index was above 0.73, showing a pleasing discriminative ability to provide patients with prognostic information in a personalized manner. Likewise, AUC also implies good discriminative ability. The calibration curve shows that the predicted values of the nomogram have a high agreement. In addition, DCA was performed to provide the clinical net benefit of the predictive model. In this study, all results indicated that the DCA curves of the 1-, 3-, and 5-year OS/CSS rates of the new model yielded a significant net clinical benefit. Our calculator can easily and quickly calculate the risk grouping of MCC patients by inputting clinically relevant characteristics.

This study still had some limitations. Firstly, the population data provided by the SEER database comes from a portion of the Caucasian population, which leads to racial limitations. As we mentioned, we need more complete ethnic data to complete the relevant research. Secondly, our prognostic risk factors were still insufficient. Due to database limitations, we lacked pathologically relevant features of MCC. If vitamin D deficiency, immunosuppressed status, MCPyV infection, CD8+ T cell levels, lymphovascular invasion, tumor growth pattern, and other information can be combined into the nomogram, the prediction of the nomogram will be more accurate and more individual (9–12). Thirdly, as mentioned above, we lack population data of new treatments and only include classical treatment methods for analysis. At present, there is a lack of large sample data to test the long-term effectiveness of immunotherapy. If immunotherapy and targeted molecular therapy can be used to analyze prognosis in future research, it will be of great benefit to the treatment progress of MCC. Finally, although we performed external validation, we did not analyze 5-year survival due to time constraints.

## Conclusion

In conclusion, we combined demographic and clinicopathological characteristics from the SEER database to build efficient nomograms to predict prognostic factors in MCC patients. Among them, advanced age, male, black race, head and neck, lymph nodes involvement, AJCC stage, no surgery, and no radiotherapy were all associated with poor outcomes. The nomograms we established can well combine relevant risk factors to predict the 1-, 3-, and 5-year OS/CSS rates of MCC patients. We established a calculator which can easily and quickly calculate the risk grouping of MCC patients by inputting clinically relevant characteristics. For patients in the high-risk group, it is recommended to shorten the follow-up interval, and timely pay attention to whether recurrence, lymph node metastasis, and distant metastasis occur, which is of great significance for improving the prognosis of patients.

## Data availability statement

The original contributions presented in the study are included in the article/supplementary material. Further inquiries can be directed to the corresponding author.

## Ethics statement

This study was undertaken without institutional review board approval or informed consent since the SEER database is publicly accessible.

## Author contributions

WX and JZ designed the study. YL was in charge of data collection and processing. The manuscript was written by WX and YL and was evaluated and modified by JZ. All authors contributed to the article and approved the submitted version.

## Acknowledgments

We’d like to express our gratitude to the SEER database for allowing us to access free and open data.

## Conflict of interest

The authors declare that the research was conducted in the absence of any commercial or financial relationships that could be construed as a potential conflict of interest.

## Publisher’s note

All claims expressed in this article are solely those of the authors and do not necessarily represent those of their affiliated organizations, or those of the publisher, the editors and the reviewers. Any product that may be evaluated in this article, or claim that may be made by its manufacturer, is not guaranteed or endorsed by the publisher.
